# A Cross-Sectional Study of Emergency Care Services During the COVID-19 Pandemic: A Multicenter Study of Healthcare Staff Perspectives

**DOI:** 10.7759/cureus.61475

**Published:** 2024-06-01

**Authors:** Ahmet Bütün, Yeşim Yeşil

**Affiliations:** 1 Department of Nursing, Faculty of Health Sciences, Mardin Artuklu University, Mardin, TUR; 2 Department of Midwifery, Faculty of Health Sciences, Mardin Artuklu University, Mardin, TUR

**Keywords:** pandemic, covid-19, healthcare system, healthcare staff, emergency care, emergency department

## Abstract

Introduction: The coronavirus disease 2019 (COVID-19) pandemic has significantly impacted various aspects of healthcare services, including emergency care services. Healthcare staff face mental issues and physical exertion when caring for patients potentially infected with COVID-19. Understanding the experiences and perspectives of emergency department (ED) healthcare staff during the COVID-19 pandemic is essential to inform evidence-based interventions and strategies to mitigate the impact on emergency care services. This study aims to investigate the experiences of ED healthcare staff regarding emergency care services during the COVID-19 pandemic, thus providing valuable insights into the challenges faced.

Materials and methods: This study utilized a cross-sectional study design. Data were collected from 256 ED healthcare staff working in nine different hospitals located in Turkey between November 15, 2021, and December 30, 2021. Data were analyzed using descriptive statistics.

Results: A total of 256 participants were included in the study. Of the participants, 58.6% were nurses, 19.5% were ED doctors, and 21.9% were emergency medical technicians. In addition, 67.2% of the participants were infected with COVID-19, and almost all of them (94.1%) were psychologically affected by the pandemic process. It was found that 85.2% of ED healthcare staff felt excluded by society due to being healthcare staff and 71.9% had to be separated from their families. Nurses were separated from their families at the highest rate (78%) during this period.

Conclusion: More than half of the ED healthcare staff had problems accessing protective equipment and were separated from their families during the pandemic due to the risk of COVID-19 transmission. Although the number of ED visits decreased because of restrictions at the beginning of the pandemic, ED visits increased again with the abolition of restrictions.

## Introduction

Coronavirus disease 2019 (COVID-19) was declared a pandemic by the World Health Organization (WHO) on March 11, 2020. Subsequently, preventing the spread of the pandemic has been the primary goal across the world. A wide variety of individual protection measures have been implemented worldwide. Both the WHO and health authorities have made some recommendations (e.g., hand washing, avoiding crowded areas, observing social distancing, and using face masks) to protect the public against this droplet-borne disease and reduce its spread [1].

The COVID-19 pandemic has significantly impacted various aspects of healthcare services, including emergency care services. Healthcare institutions, especially emergency departments (EDs), where the first contact with the patient is made, have risky environments, and healthcare staff have the highest probability of getting an infection during a pandemic. Healthcare staff have been at the forefront of managing the challenges posed by the pandemic, and understanding their perspectives is crucial for developing effective strategies to address the evolving healthcare needs.

EDs are the first units to which all patients seek care during a pandemic; therefore, the workload of ED staff has increased significantly. The behavior of patients who visit the ED, which is an important element of the healthcare system, is affected by external changes caused by natural disasters and pandemics. It has been reported that healthcare staff working in the ED, intensive care units, and isolation wards are more vulnerable to the development of adverse psychiatric effects compared with staff in other departments, possibly due to being exposed to infected patients [2].

Healthcare staff face mental problems and physical exertion when caring for patients potentially infected with COVID-19. The COVID-19 pandemic has continued, and long-term effects on mental health remain unknown [3]. The COVID-19 pandemic has raised concerns about the mental health of healthcare staff and job satisfaction [4], highlighting the need for comprehensive support systems to address the well-being of healthcare professionals. Understanding the experiences and perspectives of ED healthcare staff during the COVID-19 pandemic is needed to inform evidence-based interventions and strategies to mitigate the impact on emergency care services. This multicenter study aims to investigate the experiences of ED healthcare staff regarding emergency care services during the COVID-19 pandemic, thus providing valuable insights into the challenges faced.

## Materials and methods

Study design

This study utilized a cross-sectional design to gather data from ED healthcare staff in Turkey.

Participants and settings

The participants of this study were ED healthcare staff working in nine different hospitals, including Mardin Education and Research Hospital, Kiziltepe Public Hospital, Midyat Public Hospital, Nusaybin Public Hospital, Dargecit Public Hospital, Savur Public Hospital, Omerli Public Hospital, Mazidagi Public Hospital, and Derik Public Hospital, which are all located in the southeast of Turkey. This study included ED doctors, ED nurses, and emergency medical technicians. The convenience sampling method was utilized to recruit participants. A total of 374 ED healthcare staff are employed in nine hospitals. In a study where the population is 374 persons, it is calculated that at least 190 persons should be included in the sample for the sample to represent the population with a 95% confidence interval and 5% error rate. This study was conducted with 256 ED healthcare staff. The stratified sampling method was employed. Further details are provided in Table 1.

**Table 1:** Settings, total number of ED healthcare staff, and recruited number of ED healthcare staff ED: emergency department

Settings	Total number of ED staff (number)	Number of recruited ED staff (number)	%
Mardin Education and Research Hospital	95	65	25.4
Kiziltepe Public Hospital	70	48	18.7
Midyat Public Hospital	47	32	12.6
Nusaybin Public Hospital	44	30	11.8
Mazidagi Public Hospital	24	16	6.4
Derik Public Hospital	22	15	5.9
Savur Public Hospital	26	18	7
Dargecit Public Hospital	24	16	6.4
Omerli Public Hospital	22	15	5.9
Total	374	256	100

Data collection

An online survey using Google Forms (Google, Inc., Mountain View, CA) was conducted with 256 ED staff regarding their experiences in the ED during the COVID-19 pandemic between November 15, 2021, and December 30, 2021. A questionnaire form was developed by the researchers (AB and YY) and then tested with three healthcare staff and revised based on their feedback. The questionnaires asked participants to recall their experiences from the onset of the pandemic in March 2020. The questionnaires were filled out by the researchers (AB and YY) by asking the questions to the participants and receiving their answers face-to-face. Following such an approach allowed to collect reliable data. Any information identifying the participants was not asked, thus ensuring confidentiality and anonymity.

Data analysis

Data were analyzed using SPSS for Windows version 25.0 (IBM SPSS Statistics, Armonk, NY). While analyzing the data, descriptive statistics (number and percentage distributions) were applied in the analysis of the findings for the demographic characteristics of the participants, and the chi-square test was applied in the evaluation of the relationship between the variables. The level of statistical significance was set as p<0.05.

Ethical considerations

The required ethical approval was obtained from the Non-interventional Clinical Research Ethics Committee of Mardin Artuklu University to conduct this study (date: 01/11/2021, reference number: E-76272411-900-33470). Participants were informed about the aim of the study, and verbal consent was obtained from them. This study followed the principles of the Declaration of Helsinki.

## Results

More than half (51.2%) of the ED healthcare staff were aged between 21 and 30 years, 23% were high school graduates, 55.1% were undergraduates, and 21.9% were postgraduates. When examining the professions of the participants, 58.6% of them were nurses, 21.9% were emergency medical technicians, and 19.5% were ED doctors. It was determined that 46.9% of the participants had 0-5 years of working experience in the ED, 41.8% of them had 6-10 years, 5.9% of them had 11-15 years, and 5.5% had 16 years or more. Further characteristics of the participants are provided in Table 2.

**Table 2:** Sociodemographic characteristics of the participants ED: emergency department

Variables		Number	%
Age (years)	21-30	131	51.2
	31-40	108	42.2
	41+	17	6.6
Occupation	ED doctor	50	19.5
	Nurse	150	58.6
	Emergency medical technician	56	21.9
Education level	High school	59	23
	Bachelor's degree	141	55.1
	Master's degree	56	21.9
Years of experience in the ED	0-5 years	120	46.9
	6-10 years	107	41.8
	11-15 years	15	5.9
	16+	14	5.5
Total		256	100

The opinions of the ED staff participating in this study about the COVID-19 pandemic are provided in Table 3.

**Table 3:** ED staff views on the COVID-19 pandemic ED: emergency department, COVID-19: coronavirus disease 2019

Variables			Number	%
Status of infection with COVID-19		Yes	172	67.2
		No	84	32.8
Psychological impact of the COVID-19 pandemic on the staff		Yes	241	94.1
		No	2	0.8
		Partly	13	5.1
Feeling excluded by society due to being a healthcare staff member during the COVID-19 pandemic		Yes	218	85.2
		No	14	5.5
		Partly	24	9.3
Separation from your family during the COVID-19 pandemic		Yes	184	71.9
		No	11	4.3
		Partly	61	23.8
Feeling that you have been adequately supported by your organization during the COVID-19 pandemic		Yes	21	8.2
		No	106	41.4
		Partly	129	50.4
Provided information by your organization on how to prepare for the COVID-19 pandemic		Yes	201	78.5
		No	22	8.6
		Partly	33	12.9
Sharing updated guidelines on the operation of the ED due to the COVID-19 pandemic		Yes	204	79.7
		No	19	7.4
		Partly	33	12.9
Publishing information materials by your institution or by the Ministry of Health in relation to managing patients in the ED during the COVID-19 pandemic		Yes	210	82
		No	15	5.9
		Partly	31	12.1
Changes in your workplace at the beginning of the COVID-19 pandemic		Yes	98	38.3
		No	158	61.7
		Partly	10	3.9
Providing satisfactory answers to the questions of patients admitted to the ED		Yes	19	7.4
		No	95	37.1
		Partly	142	55.5
Considering that organizations related to the health sector have taken the necessary initiatives and studies on the issues and problems that concern healthcare professionals during the pandemic		Yes	30	11.7
		No	107	41.8
		Partly	119	46.5
Adequate provision of protective equipment in the ED during the COVID-19 pandemic	Glove	Yes	225	87.9
		No	9	3.5
		Partly	22	8.6
	Mask	Yes	219	85.5
		No	6	2.3
		Partly	31	12.1
	Apron	Yes	91	35.5
		No	38	14.9
		Partly	127	49.6
	Visor	Yes	111	43.4
		No	22	8.6
		Partly	123	48
Believing that adequate work has been done to minimize the risk of transmission in the ED		Yes	8	3.1
		No	130	50.8
		Partly	118	46.1
ED visits during the COVID-19 pandemic	Total number of visits to the ED	Decreased	43	16.8
		No change	106	41.4
		Increased	107	41.8
	Number of "non-urgent" visits to the ED	Decreased	24	9.4
		No change	97	37.9
		Increased	135	52.7
	Number of "non-urgent" visits to the ED after the abolition of restrictions	Decreased	4	1.6
		No change	4	1.6
		Increased	248	96.8

Of the participants, 67.2% of ED staff stated that they had COVID-19, and almost all of them (94.1%) stated that they were psychologically affected by the pandemic. It was found that 85.2% of the participants felt excluded by society due to being healthcare staff and 71.9% had to be separated from their families. During the COVID-19 pandemic, 50.4% of them were partially supported by their institution, while 41.4% stated that they thought they were not supported enough. Of the ED staff, 78.5% reported that they were informed by their institution about how to prepare for this process, 79.7% reported that up-to-date guidelines on the situation were shared, and 82% reported that information materials were shared with them. In addition, most of the ED staff (82%) stated that they read up-to-date information on COVID-19. In addition, 38.7% of the ED staff have experienced workplace change since the beginning of the COVID-19 pandemic. In addition, 55.5% of the ED staff stated that they were able to partially answer patients' questions about the process. In addition, 46.5% of the participants thought that health sector organizations were partially interested in the issues and problems that concern health professionals in the COVID-19 pandemic, and 41.8% of them thought that the related organizations were not interested in such issues. In this pandemic, participants stated that they were supplied with gloves (87.6%), masks (85.5%), aprons (35.5%), and visors (43.4%). Half of the ED staff (50.8%) did not believe that the necessary work was being done to reduce the risk of transmission in the ED. Of the ED staff, 41.8% stated that the total ED visits increased, 52.7% of them stated that non-urgent ED visits increased, and 96.8% of them stated that there was an increase in non-urgent ED visits after the abolition of restrictions.

The relationship between some variables related to the opinions of the ED healthcare staff participating in this study about the COVID-19 pandemic and their professions is given in Table 4.

**Table 4:** Examination of some variables in terms of the profession of ED healthcare staff *p<0.05, **chi-square analysis ED: emergency department, COVID-19: coronavirus disease 2019

Variables			ED doctors		Nurses		Emergency medical technicians		Test value	P value
			Number	%	Number	%	Number	%		
Considering that organizations related to the health sector have taken the necessary initiatives and studies on the issues and problems that concern healthcare professionals during the pandemic		Yes	2	4	18	12	10	17.9	16.485**	0.002*
		No	14	28	74	49.3	19	33.9		
		Partly	34	68	58	38.7	27	48.2		
Status of infection with COVID-19		Yes	26	52	114	76	32	57.1	13.078**	0.001*
		No	24	48	36	24	24	42.9		
Separation from your family during the COVID-19 pandemic		Yes	34	68	117	78	33	58.9	11.672**	0.020*
		No	1	2	8	5.3	2	3.6		
		Partly	15	30	25	16.7	21	37.5		
Feeling that you have been adequately supported by your organization during the COVID-19 pandemic		Yes	0	0	16	10.7	5	8.9	18.740**	0.001*
		No	16	32	74	49.3	16	28.6		
		Partly	34	68	60	40	35	62.5		
Provided information by your organization on how to prepare for the COVID-19 pandemic		Yes	47	94	106	70.7	48	85.8	15.106**	0.004*
		No	0	0	18	12	4	7.1		
		Partly	3	6	26	17.3	4	7.1		
Changes in your workplace at the beginning of the COVID-19 pandemic		Yes	23	46	64	42.7	11	19.6	10.716**	0.005*
		No	27	54	86	57.3	45	80.4		
Sharing updated guidelines on the operation of the ED due to the COVID-19 pandemic		Yes	47	94	110	73.3	47	83.9	11.505**	0.021*
		No	0	0	16	10.7	3	5.4		
		Partly	3	6	24	16	6	10.7		
Adequate provision of protective equipment in the ED during the COVID-19 pandemic	Glove	Yes	50	100	132	88	43	76.8	20.944**	0.001*
		No	0	0	8	5.3	1	1.8		
		Partly	0	0	10	6.7	12	21.4		
	Mask	Yes	50	100	129	86	40	71.4	20.676**	0.001*
		No	0	0	5	3.3	1	1.8		
		Partly	0	0	16	10.7	15	26.8		
	Apron	Yes	29	58	53	35.3	9	16.1	28.587**	0.001*
		No	5	10	16	10.7	17	30.4		
		Partly	16	32	81	54	30	53.5		
	Visor	Yes	39	78	56	37.3	16	28.6	32.314**	0.001*
		No	1	2	16	10.7	5	8.9		
		Partly	10	20	78	52	35	62.5		
Total			50	100	150	100	56	100		

There was a significant relationship between their occupation and status of being infected with COVID-19 (p<0.05). Of the ED healthcare staff who had COVID-19, 76% were nurses. In this pandemic process, there was a significant relationship between separation from their families, being supported by the institution, and providing information about pandemic preparedness in the process and professions (p<0.05). Nurses were separated from their families at the highest rate (78%) during the pandemic. It was determined that there was a significant relationship between occupations and workplace change (p<0.05), while other healthcare staff experienced the least workplace change (19.6%). There was a significant difference between the sharing of updated guidelines on the functioning of EDs and the professions (p<0.05). There was a significant difference between the provision of gloves, masks, aprons, and visors as protective equipment and professions (p<0.05). Almost every professional group reported that gloves and masks were provided. However, more than half of the nurses and emergency medicine technicians stated that the supply of aprons and visors was partially available. There is a significant relationship between health organizations working during the COVID-19 pandemic and professions (p<0.05), with more than half of doctors (68%) believing that studies have been conducted.

The relationship between the years of work experience of ED staff participating in the study and some variables related to the COVID-19 pandemic is provided in Table 5.

**Table 5:** Examination of some variables related to the COVID-19 pandemic in relation to years of work experience of ED healthcare staff *p<0.05, **chi-square analysis ED: emergency department, COVID-19: coronavirus disease 2019

Variables			0-5 years		6-10 years		11-15 years		16+ years		Test value	P value
			Number	%	Number	%	Number	%	Number	%		
Sharing updated guidelines on the operation of the ED due to the COVID-19 pandemic		Yes	93	77.5	91	85	10	66.7	10	71.4	13.448**	0.036*
		No	12	10	7	6.6	0	0	0	0		
		Partly	15	12.5	9	8.4	5	33.3	4	28.6		
ED visits during the COVID-19 pandemic	Total number of ED visits	Decreased	22	18.3	16	15	4	36.7	1	7.1	24.663**	0.001*
		No change	32	26.7	57	53.3	9	60	8	57.1		
		Increased	66	55	34	31.8	2	13.3	5	35.8		
	"Non-urgent" ED visits	Decreased	16	13.3	5	4.7	2	13.3	1	7.1	23.985**	0.001*
		No change	30	25	50	46.7	11	73.4	6	42.9		
		Increased	74	61.7	52	48.6	2	13.3	7	50		
Total			120	100	107	100	15	100	14	100		

There was a significant relationship between the status of health organizations sharing information about the pandemic and current guidelines on the functioning of ED services and their years of work experience (p<0.05). During the pandemic, there was a significant relationship between total ED visits and non-urgent visits, and years of work experience of ED staff (p<0.05), and more than half of the participants with 0-5 years of work experience stated that both total ED visits and non-urgent ED visits increased during the COVID-19 pandemic.

## Discussion

This study aimed to investigate emergency care services during the COVID-19 pandemic from the perspective of healthcare staff. Most participants in the study were nurses, and more than half of them have an age range of 21-30 years. It was determined that the average number of years of working experience in the ED was mostly between 0 and five years. In some studies conducted in different countries, it has been reported that the number of years of working experience was between five and 10 years [5]. The reason for differences in years of working experience could be institutional policy, which aimed to appoint newly graduated nurses, doctors, and emergency medical technicians to gain experience in the busiest services.

Frontline healthcare staff are more likely to become infected with the virus because they provide direct care to patients with COVID-19 [6,7]. This study found that more than half of the ED staff (67.2%) had been infected with COVID-19. Also, the COVID-19 pandemic process negatively affected the psychology of almost all ED healthcare staff (94.1%). In a study conducted on physicians working in the ED at the beginning of the COVID-19 pandemic in Turkey, the prevalence of anxiety was found to be 35.5% and that of depression was found to be 62% among physicians [8]. Frontline healthcare staff who directly provide care to patients with COVID-19 have been reported to be at a higher risk of depression, anxiety, insomnia, and distress [9]. In particular, high rates of depression/anxiety and burnout were observed in individuals working in an environment with few resources and little perceived organizational support [10].

Factors such as excessive workload, fear of infecting family members, and death of healthcare staff can also cause stress and anxiety [11,12]. In line with the existing literature, COVID-19 is believed to be highly contagious, causing frontline healthcare staff to feel anxious and stressed about their family members [13,14]. The existing literature confirmed the results of this study and showed that there was a psychological effect related to the COVID-19 pandemic process [15]. During the pandemic, it was determined that it was difficult for hospital employees to return home because they were worried that their family members would be infected with the virus [16]. According to the results of this study, 71.9% of the ED staff stated that they had to be separated from their families during the pandemic. Severe acute respiratory syndrome coronavirus 2 (SARS-CoV-2) has been shown to be predominantly spread through direct, indirect, or close contact with infected respiratory droplets [17,18]. Healthcare staff have isolated themselves to protect their family members from this pandemic.

According to the results of this study, most of the ED staff (82%) reported that the hospital or the Ministry of Health had published information materials covering the COVID-19 pandemic aimed at managing patients in ED services. Being prepared is crucial to minimize the impact of pandemics and large-scale public health emergencies on patients, healthcare staff, and health systems. Therefore, emergency and disaster management response plans must be in place. The first preparation plan for the COVID-19 pandemic in Turkey was planned by the Ministry of Health within the framework of the Pandemic Influenza National Preparedness Plan [19]. Research has shown that healthcare staff feel better prepared for pandemics if they have access to up-to-date, consistent, and clear information [16]. It has been noted that if the information is inconsistent and unclear, it results in stress, confusion, and insecurity [20]. The results showed that there was a difference in the comparison between the institutional management's information about how to prepare for the COVID-19 pandemic and the occupation groups (p<0.05). Hospitals, especially ED services, must find practical strategies to manage potential waves during outbreaks and prevent further transmission [21]. The healthcare organization must transparently develop professional guidelines and directives for patient care to ensure trust and commitment to emerging clinical information. Government and public health initiatives should foster a greater sense of solidarity and trust among healthcare staff [22].

When examining how ED healthcare staff were provided with protective equipment during the COVID-19 pandemic, ED healthcare staff were provided with gloves (87.6%), masks (85.5%), aprons (35.5%), and visors (43.4%). The use of appropriate protective equipment to prevent and limit the spread and transmission of COVID-19 to healthcare staff has been strongly recommended [23,24]. Stocking of protective equipment and access to it has been a critical issue throughout the COVID-19 pandemic [16]. Our study results also show that there is a difference between professions in terms of access to protective equipment. Doctors had greater access to aprons and visors than nurses and emergency medical technicians. This indicates that aprons and visors were not as adequate or easily accessible as other protective equipment. This has been a cause for concern for ED staff during the pandemic.

According to the results of this study, 41.8% of the ED staff stated that total ED visits increased, 52.7% of ED staff stated that non-urgent visits to the ED increased, and 96.8% of ED staff stated that there was an increase in non-urgent visits to the ED after the abolition of restrictions. In some studies conducted in Turkey and various other countries, it was determined that there was a decrease in the number of ED visits during the COVID-19 pandemic process [25-28]. The reasons for the reduction in the number of ED visits during the pandemic period could be as follows: the distance between people due to the lockdown, travel and activity restrictions and the consequent reductions in non-COVID-19 respiratory infectious diseases, the decrease in accidents and injuries because of the same restrictions, the effort by the public to reduce the pressure on the health system because of the increase of public awareness through the media, keeping patients with COVID-19 and their contacts at home, the establishment of call centers that can guide patients on choosing the most appropriate health services for their care needs, and the fear of infection transmission that may occur in the ED.

Apart from the pandemic, compared with Europe and Organisation for Economic Co-operation and Development (OECD) countries, Turkey was found to be the country with the highest number of ED visits by population [29]. As emphasized in this study, ED staff stated that the number of ED visits has increased with the abolition of restrictions. Inappropriate ED visits lead to a loss of time in the medical team, an increase in workload, and a decrease in attention, and are an obstacle to giving due time and attention to those in need of urgent care [30].

Clinical and research implications

This study could provide a better understanding of how ED staff were affected by the COVID-19 pandemic, including the psychological effects on ED staff and related difficulties. Developing appropriate interventions to minimize the difficulties faced during COVID-19 would allow better management of future pandemics. This study could inform researchers, ED directors, and policymakers regarding the impact of the COVID-19 pandemic on emergency care services. Future research could focus on improving the psychological well-being of ED healthcare staff to better deal with future pandemics.

Strengths and limitations

A strength of the study is the collection of data from nine different hospitals. One of the limitations of the study could be collecting data around 18 months after the onset of the pandemic, and this could affect how ED healthcare staff recall their experiences. However, difficulties in recalling their experiences were not reported by ED healthcare staff. It is also important to state that there were lockdown measures and it was impossible to conduct any study by collecting data face-to-face before the data collection period of this study.

## Conclusions

This study evaluated emergency care services during the COVID-19 pandemic from the perspective of healthcare staff. It has been concluded that healthcare staff working in the ED have problems accessing protective equipment, that they have to be separated from their families during the pandemic process due to the risk of COVID-19 transmission, and that more than half of them have been infected with COVID-19. In addition, as frontline healthcare staff, almost all were psychologically affected by the COVID-19 pandemic. Although the number of ED visits decreased during the COVID-19 pandemic, ED visits increased again with the abolition of restrictions. This study provides evidence to enable healthcare authorities to identify how COVID-19 affects the emergency care services and ED healthcare staff, take precautions for the difficulties faced during the pandemic, enhance the resilience of healthcare systems for future crises, and thus better manage future pandemics.
